# Online panels in social science research: Expanding sampling methods beyond Mechanical Turk

**DOI:** 10.3758/s13428-019-01273-7

**Published:** 2019-09-11

**Authors:** Jesse Chandler, Cheskie Rosenzweig, Aaron J. Moss, Jonathan Robinson, Leib Litman

**Affiliations:** 1 University of Michigan, Ann Arbor, MI USA; 2 Columbia University, New York, NY USA; 3 Prime Research Solutions, Queens, NY USA; 4 Lander College, Kew Gardens Hills, New York, NY USA

**Keywords:** Mechanical Turk, Prime panels, Online experimentation, Data collection

## Abstract

Amazon Mechanical Turk (MTurk) is widely used by behavioral scientists to recruit research participants. MTurk offers advantages over traditional student subject pools, but it also has important limitations. In particular, the MTurk population is small and potentially overused, and some groups of interest to behavioral scientists are underrepresented and difficult to recruit. Here we examined whether online research panels can avoid these limitations. Specifically, we compared sample composition, data quality (measured by effect sizes, internal reliability, and attention checks), and the non-naivete of participants recruited from MTurk and Prime Panels—an aggregate of online research panels. Prime Panels participants were more diverse in age, family composition, religiosity, education, and political attitudes. Prime Panels participants also reported less exposure to classic protocols and produced larger effect sizes, but only after screening out several participants who failed a screening task. We conclude that online research panels offer a unique opportunity for research, yet one with some important trade-offs.

**Electronic supplementary material:**

The online version of this article (10.3758/s13428-019-01273-7) contains supplementary material, which is available to authorized users.

The use of online participant recruitment practices is one of the most significant changes in the social and behavioral sciences in the last 20 years (for a historical overview, see Gosling & Mason, 2015). Online recruitment provides an affordable way to reach participants outside the university community, making it possible to recruit samples that more closely reflect the diversity of the US population or to selectively recruit hard to reach samples of participants. College students—and the communities they tend to live in—differ from the US population as a whole in important ways, including education, political ideology, religious affiliation, and racial and ethnic diversity (Berinsky, Huber, & Lenz, 2012; Gosling, Sandy, John, & Potter, 2010; Henrich, Heine, & Norenzayan, 2010; Pew Research Center, 2016b, 2017). The use of college student samples has also been criticized because students are young and relatively inexperienced with the world, making their judgments and attitudes fundamentally different from those of older adults (Jones & Sonner, 2001; Sears, 1986).

Although it has been technically possible to use the internet to recruit research participants for a long time, the ability to do so simply and cost-effectively is relatively new. Not so long ago, researchers were faced with one of two choices: they could recruit participants themselves or they could contract the work out to a survey sample provider like a polling or market research company. Researchers who opted to recruit participants themselves had to overcome a string of technical challenges including where to find people, how to verify their identity, and how to securely pay them. Contracting the work out was slow and inflexible, with contract negotiations adding weeks or months to data collection times and sample providers placing constraints on sample size and survey length (for an overview of these challenges, see Craig et al., 2013). In many cases, the surveys were programmed and deployed on the survey company’s own platform, adding considerably to cost.

Two parallel developments made it easier for researchers to recruit their own participants. Survey software companies (such as Qualtrics and SurveyMonkey) simplified the programming and fielding of web surveys. At about the same time, Amazon’s Mechanical Turk (MTurk) simplified participant recruitment. To do so, MTurk established a common marketplace in which researchers and research participants could find each other, a reputation system to eliminate bad actors (Peer, Vosgerau, & Acquisti, 2014), and a secure means of paying participants. These features addressed many of the difficulties faced by researchers collecting samples for themselves, without the inflexibility or cost of contracting out the entire data collection process.

In many ways, MTurk performs well as a recruitment tool. It is able to quickly deliver many participants (hundreds per day) at a low cost (between $0.10 and $0.25 per minute of participant time). The quality of the data provided by MTurk samples is also quite high, typically equaling that obtained from traditional college student samples (Buhrmester, Kwang, & Gosling, 2011; Farrell, Grenier, & Leiby, 2017; Goodman, Cryder, & Cheema, 2013; Horton, Rand, & Zeckhauser, 2011; Litman, Robinson, & Rosenzweig, 2015; Paolacci & Chandler, 2014; Shapiro, Chandler, & Mueller, 2013). For these reasons, MTurk has revolutionized behavioral research, with hundreds of peer-reviewed articles being published each year that rely on the MTurk participant pool (see Chandler & Shapiro, 2016; Goodman & Paolacci, 2017; Stewart, Chandler, & Paolacci, 2017).

For all MTurk’s strengths, however, it also has limitations that might be unacceptable to some researchers. First, the pool of available workers is actually smaller than might be assumed from its 500,000 registered users. Although the actual number of workers remains to be determined, studies generally agree that the number of workers a researcher can access at any particular time is at least an order of magnitude lower than the number of registered users (Difallah, Filatova, & Ipeirotis, 2018; Stewart et al., 2015; Robinson, Rosenzweig, Moss, & Litman 2019). A natural consequence of a small participant population and a large researcher population is that participants complete many—sometimes related—studies. This creates concerns that prior exposure to research materials (“non-naivete”) can compromise data quality (Chandler, Paolacci, Peer, Mueller, & Ratliff, 2015; DeVoe & House, 2016; Rand et al., 2014; but see also Bialek & Pennycook, 2018; Zwaan et al., 2017).

A second concern is that although MTurk is often celebrated as offering more diverse samples than college student subject pools, this is true primarily because college student samples are extremely homogeneous. Despite increased diversity, MTurk workers still look very little like the US population. MTurk workers are overwhelmingly young, with 70% of the MTurk population being below the age of 40, as compared to just 35% in the United States as a whole. Furthermore, there are very few participants above age 60 on MTurk. Reflecting differences in age and birth cohort, MTurk samples are also more liberal, better educated, less religious, and single without children, when compared to the US population (Casey, Chandler, Levine, Proctor, & Strolovitch, 2017; Huff & Tingley, 2015; Levay, Freese, & Druckman, 2016).

Although MTurk is currently the most popular online recruitment platform, it is not the only one available to researchers. At about the same time that MTurk emerged as a source for online participant recruitment, online panels—a source of online participants for the market research industry—grew into a multibillion-dollar industry and worked to improve and diversify the products they offer (Callegaro, Villar, Yeager, & Krosnick, 2014; Rivera, 2015). Much of their effort has been focused on meeting the needs of large market research companies, who often want to reach extremely specific samples. For this reason, online panels must be able to access many diverse people and to ensure clients that their desired sample has been reached. Through a combination of active recruitment efforts, partnerships, and selective purchasing of access to competitors’ samples, online research panels have access to tens of millions of respondents (SurveyMonkey, 2017) though the same caveats about the size of MTurk's registered user base apply here, too (Hillygus, Jackson, & Young, 2014).

Given the size and focus of online panels, they have two potential advantages over MTurk. First, they are likely much more diverse. Second, the participants in online panels are probably less familiar with behavioral science studies, both because of the constant influx of new participants and because such panels are rarely used for academic studies. In addition to these advantages, some of the hassles of accessing online panels have disappeared over time. Although academic research is a small piece of the larger market for online samples, some firms have embraced the “do-it-yourself” approach to research favored by academics and epitomized by MTurk. These firms now offer more flexibility in sample recruitment (such as allowing researchers to collect data using the survey platform of their choice) and even offer samples as vendors rather than as contractors. Whereas some of these firms (such as Qualtrics and SurveyMonkey) provide samples to complement their survey platform business, others such as Prime Panels function as standalone recruitment services, with automated do-it-yourself study setup that in many ways resembles the MTurk "do-it-yourself" interface.

The increasing simplicity of using online research panels makes it worth reconsidering their use as a source of participants for academic research. However, if academics are going to use online research panels, one serious limitation must be overcome: data quality. Despite the effort of panel providers, the samples recruited from online research panels typically yield low-quality data. This might be because participants who are aggressively recruited to participate in research are unmotivated or inexperienced at completing surveys. Alternatively, the methods that sample providers use to discover bad actors might be less efficient than the reputation system used by MTurk. Regardless of the reason, studies have consistently shown that participants from online research panels are less attentive and have lower-scale reliabilities than MTurk workers (Kees, Berry, Burton, & Sheehan, 2017; Thomas & Clifford, 2017).

Fortunately, data quality problems can be addressed through careful study design. In particular, participants who produce poor-quality data can be identified and removed from the sample. Online research panels frequently determine study eligibility by using participant responses to screening questions, and researchers are not expected to pay for participants who fail to meet prespecified criteria. By including measures to identify low-quality respondents as part of the screening process, such participants can be avoided, potentially addressing the data quality issues inherent to the platform. Prime Panels, a compilation of online research panels (Ballew, Goldberg, Rosenthal, Gustafson, & Leiserowitz, 2019; Job, Sieber, Rothermund, & Nikitin, 2018; Davidai, 2018; Waggoner, 2018, Deri, Davidai, & Gilovich, 2017), includes such screening measures as part of its standard participant recruitment process. We explored the viability of this approach by comparing the data quality, demographic diversity, and participant naivete observed in samples obtained from Prime Panels and from MTurk.

## Introduction

Because data quality is a known issue for online research panels, we included a basic language comprehension screener at the beginning of the study. We compared the quality of data produced by the MTurk sample with that produced by Prime Panels participants who did and did not pass the screener. Our definition of data quality was multi-faceted. We examined not only the pass rate of attention checks, but also the internal reliabilities of validated scales, and the effect sizes of several classic psychological phenomena including the impact of gain versus loss framing (Tversky & Kahneman, 1981), the impact of anchors on numeric estimates (Jacowitz & Kahneman, 1995), and differences in moral reasoning under personal and impersonal circumstances (Hauser, Cushman, Young, Kang-Xing Jin, & Mikhail, 2007). Each measure of data quality was chosen either because it had been used to assess data quality on Mechanical Turk in the past (e.g., the Big Five Personality Inventory; Buhrmester et al., 2011) or because the experimental manipulations were known to produce strong enough effect sizes that we could conclude any lack of effect was likely due to the sample rather than the manipulation (e.g., framing effects, anchoring effects, and the trolley dilemma). Across all measures of data quality, we expected participants on Prime Panels who passed the initial screener to perform at similar levels to high-reputation MTurk workers.

### Participant diversity and representativeness

We compared the demographic characteristics of the Prime Panels and MTurk samples to that of the American National Electoral Study (ANES), which uses a high-quality probability sample of the US population. We also examined whether studies that have difficulty replicating on MTurk because of their dependence on underrepresented demographic characteristics would replicate successfully on Prime Panels. Although effects observed on MTurk usually replicate in nationally representative samples (Berinsky et al., 2012; Clifford, Jewell, & Waggoner, 2015; Coppock & McClellan, 2019; Mullinix, Leeper, Druckman, & Freese, 2015), some studies do not, probably because they are moderated by demographic characteristics that vary between MTurk and the population as a whole (Krupnikov & Levine, 2014). For example, Americans become more pro-life when they are first asked to consider God’s views on abortion (Converse & Epley, 2007), but this finding does not replicate on MTurk (Mullinix et al., 2015), which is largely atheist (Casey et al., 2017). All of the measures we chose for examining demographic differences across platforms were selected either because they had been included in previous research that compared effects obtained on Mechanical Turk with those obtained from a nationally representative, probability-based sample of Americans (see Mullinix et al., 2015) or because they were part of the ANES study and allowed us to compare MTurk and Prime Panels to a nationally representative sample.

### Naivete

Many MTurk workers are highly active, and some have completed dozens or even hundreds of studies (Rand et al., 2014). Online research panel participants also complete many studies (Hillygus et al., 2014), but the content of the studies is different and rarely includes the kinds of stimuli used in basic behavioral science research. Thus, we expected that MTurk workers would be more familiar with the measures used in this study than would Prime Panels participants. We also explored whether prior exposure to the manipulations in this study (i.e., non-naivete) would cause the effect sizes to be smaller on MTurk than on Prime Panels.

## Method

### Participants and procedure

We recruited two samples of participants. The first sample consisted of 474 US participants from MTurk who had a 95% approval rating and at least 100 prior HITs completed. We used these worker qualifications because they are standard practice for data collection on MTurk (see Peer et al., 2014). The second sample consisted of 782 US participants from Prime Panels. We collected almost double the number of participants on Prime Panels as on MTurk because we expected several Prime Panels participants to fail our initial screener and because comparing the data quality of those who passed and failed the screener was one of our central goals. Furthermore, we collected large samples on both platforms because we wanted enough participants so as to adequately describe differences in the platform demographics. Our sample sizes were more than adequate to detect condition differences with each study manipulation and roughly in line with other studies that have investigated differences in effect sizes across research platforms (e.g., Peer, Brandimarte, Samat, & Acquisti, 2017).

All participants completed an initial screener. They then completed a survey instrument (the Big Five Personality Inventory; BFI), a performance measure (Cognitive Reflection Test; CRT), and five short experiments in a randomly assigned order. After completing each of these tasks, participants were asked if they had ever seen it previously. Finally, participants reported political attitudes and demographics. Attention-check questions were included in the BFI, political attitudes, and demographics sections of the study. All the stimuli used in this study, including the exact wording of all manipulations, instructions, and questions, were pre-registered and are available online (https://osf.io/aqxy9).

### Measures

#### Initial screener

To address the problem of participant inattentiveness in online research panels, we implemented a pre-study screener that tested participants’ attentiveness and basic English comprehension. The screener consisted of four questions that each presented a target word and asked participants to name a synonym. The target words of the screening questions were taken from the Big Five Inventory, a commonly used personality scale. For example, one question asked, “Which of the following words is most related to ‘moody’?” Because most online studies require participants to read long questionnaires and to comprehend study instructions, participants who are not familiar with basic English words are not likely to adequately follow the instructions and complete the study. These items are also likely to screen out inattentive participants who provide responses without reading the questions. A CAPTCHA question was included as a final screening item.

#### Big-Five Inventory

The BFI personality questionnaire (John, Naumann, & Soto, 2008) consists of 44 short, declarative statements such as “Is talkative.” Participants indicate whether each statement applies to them (1 = *strongly agree* to 5 = *strongly disagree*). Approximately half of the items for each trait are reverse-coded. We also added ten items that were direct antonyms of the original items. For example, “tends to be organized” was reversed to be “tends to be disorganized.” As we describe later, these items were used to examine the consistency of participants’ responses (Litman, Robinson and Abberbrock, 2015).

#### Cognitive reflection test

The CRT consists of three questions that measure the tendency to provide an intuitively compelling but incorrect response over a reflective but correct response (Frederick, 2005).

#### Trolley dilemma experiment

On the basis of a thought experiment by Thomson (1976; see also Foot, 1967/1978), participants were asked whether they would sacrifice one person to save the lives of five other people and offered four response options (1 = *Definitely not*, 2 = *Probably not*, 3 = *Probably yes*, 4 = *Definitely yes*). Participants were randomly assigned to one of two versions of the trolley dilemma. In the *classic* version, participants were asked to imagine they are driving a trolley with failed brakes, which will collide with and kill five people. Participants can save the people by turning the trolley onto another track, but this would result in killing one person. In the *footbridge* version, participants were asked to imagine that a trolley with failed brakes is heading toward five people. Participants can save the people by pushing an innocent bystander in front of the train. Numerous studies have shown that people are more willing to sacrifice one life in order to save five when doing so requires turning the train than when it requires pushing a bystander from the footbridge (e.g., Hauser et al., 2007).

#### Anchoring effect: Height of Mount Everest

Participants were asked to estimate the height of Mount Everest after being randomly assigned to either a low or high anchor condition. In the low anchor condition, participants were asked whether Mount Everest is greater or less than 2,000 feet in height. In the high anchor condition, participants were asked whether Mount Everest is greater or less than 45,000 feet. Jacowitz and Kahneman (1995) found that people exposed to the high anchor tend to provide larger estimates than people exposed to the low anchor.

#### Framing effect: The Asian disease problem

Participants imagined that the United States was preparing for an outbreak of disease. They were asked to choose between two logically identical courses of action, framed in terms of losses or gains. One course of action led to a certain number of deaths (or lives saved), and the alternative could lead to an uncertain number of deaths (or lives saved). Tversky and Kahneman (1981) found that when the outcomes were framed in terms of lives saved, people preferred the certain, safe option; yet, when outcomes were framed in terms of lives lost, people preferred the uncertain, risky option.

#### With God on our side experiment

Participants were asked about their personal view of abortion and about God’s view of abortion (1 = *completely pro-choice* to 7 = *completely pro-life*). The order of these questions was randomly assigned. Converse and Epley (2007) found that in a nationally representative sample, personal opinions became more anti-abortion following the God-centered question, but this finding failed to replicate in a sample of MTurk workers (Mullinix et al., 2015, Exp. 2), presumably because of the high proportion of atheists in the MTurk sample.

#### Public attitudes about political equality experiment

Participants were asked whether they supported political equality (1 = *Strongly support* to 5 = *Strongly oppose*). Participants were randomly assigned to answer this question either without a definition of political equality or after political equality was defined as “making sure every citizen has the right to vote and participate in politics to make their opinions known to government.” Flavin (2011) found that in a nationally representative sample, participants were significantly less likely to support political equality when it was undefined than when it was defined, but this difference was significantly smaller in a sample of MTurk workers (Mullinix et al., 2015), presumably because they are younger, more politically liberal, and more likely to endorse equality regardless of its definition.

#### Naivete

Naivete was measured after each experimental manipulation, with the following question: “You just responded to questions involving X. . . . Have you ever participated in studies that asked these questions previously (even if the wording was not exactly the same)?”

#### Political attitudes and beliefs

We included questions from the American National Election Studies Pilot and Time Series (ANES, Stanford University, & University of Michigan, 2016) that asked people about their attitudes toward minority (e.g., African Americans, Hispanics, Muslims) and majority (e.g., Whites, Christians, men) groups, whether minority and majority groups face discrimination, whether the police treat minorities worse than other groups, and their attitudes toward several specific issues, including affirmative action, the gender wage gap, capital punishment, same-sex marriage, terrorist attacks, and political correctness.

#### Demographic variables

Questions measuring demographics were taken from the ANES (ANES, Stanford University, & University of Michigan, 2016). Specifically, we asked participants questions about their gender, racial background, age, level of education, marital status, political affiliation, religion, and household income.

#### Attention check questions

Four attention checks were included in the survey. Two questions were inserted in the BFI and took the same form as the other BFI questions: One read “is not reading the questions in the survey” and the other read “is reading the questions in the survey.” The other two attention check questions were embedded within the demographic questions. The first read “Please select ‘Satisfied’ on the scale (second from the left): This item is for verification purposes” with five response options ranging from *Very satisfied* to *Not at all satisfied*. The second question read “I am not reading the questions in this survey,” with five response options (1 = *Disagree strongly* to 5 = *Agree strongly*).

## Results

### Screener questions

As predicted, the pass rate for the initial screener—defined as answering all four questions correctly—was significantly higher on MTurk (446 out of 474 participants, 94.1%) than on Prime Panels (534 out of 782 participants, 68.3%). Ordinarily, Prime Panels participants who fail prescreening measures are terminated and do not have the opportunity to complete downstream measures. In this study all participants completed all measures, so we could compare those who passed the screener to those who failed it.

For each measure of data quality that follows, we compared the responses from MTurk participants who passed the initial screener, Prime Panels participants who passed the screener, and Prime Panels participants who failed the screener. Although we originally planned to analyze the data from all MTurk participants, we realized after collecting the data that retaining only those who passed the screener would be parallel with the Prime Panels sample. Furthermore, analyses conducted with and without the MTurk participants who failed the screener showed that there were no changes in the overall pattern of results.

### Data quality measures

We examined data quality across samples using participants’ performance on attention checks, the reliability of their self-reports, the speed with which they completed the longest questionnaire in the study, and the effect sizes of three classic experimental manipulations. We also conducted exploratory analyses to examine the percentage of participants who completed the study on a mobile device, which internet browser they used, and whether they tried to reenter the study—all factors that might affect data quality. The results for these exploratory measures are reported in the supplementary materials.

#### Attention checks

MTurk participants who passed the initial screener also performed well on the attention manipulation checks. Specifically, 90% passed all four attention checks, and 98% passed three of them. Of the participants who passed the initial screener on Prime Panels, 78% passed all four attention checks, and 91.5% passed three of them. The performance of Prime Panels participants who failed the initial screener was much lower, with just 45% passing all four attention checks, and 70.6% passing three. A one-way analysis of variance (ANOVA) confirmed this pattern of performance, *F*(2, 1225) = 112.25, *p <* .001. MTurk participants performed the best (*M =* 3.86, *SD =* 0.44), followed by the Prime Panels passed (*M =* 3.66, *SD =* 0.74), and then the Prime Panels failed (*M =* 2.95, *SD =* 1.22) groups; all pairwise comparisons between groups were significant (*p*s *<* .05).

#### Reliability of self-report

We assessed the reliability of participants’ self-report by calculating omegas for the five personality factors of the BFI. As displayed in Table 1, MTurk participants had the highest reliability, followed by Prime Panels participants who passed the screener, and then Prime Panels participants who failed the screener. The reliability scores for Prime Panels participants who passed the screener were comparable to previously published data (see Buhrmester et al., 2011; John & Srivastava, 1999). In addition, the omegas for Prime Panels participants who failed the screener were .6 or lower for the conscientiousness, extraversion, and agreeableness dimensions, which are very low compared to previously published norms (e.g., John & Srivastava, 1999). The reliability scores for the current MTurk workers were higher than those observed in other samples, including earlier samples of MTurk workers (Buhrmester et al., 2011; Litman et al., 2015; see the last column of Table 1 and the alphas in Table S1 in the supplementary materials), suggesting that MTurk workers remain good at responding to long measures that require attention, such as the BFI. Finally, we conducted an analysis looking at individual-level consistency using the squared discrepancy procedure (Litman et al., 2015). Because these results were generally in line with group-level measures of reliability, we report squared discrepancy scores in section II of the supplementary materials.

**Table 1:** Omega coefficients for the dimensions of the BFI

Dimension	Sample			
	MTurk	Prime Panels Passed	Prime Panels Failed	MTurk (Litman et al., 2015)
Openness	.85 [.78, .86]	.80 [.77, .83]	.73 [.68, .76]	.83 [.75, .88]
Conscientiousness	.89 [.86, .90]	.82 [.78, .85]	.60 [.47, .69]	.84 [.75, .90]
Extraversion	.90 [.88, .92]	.83 [.80, .85]	.58 [.45, .66]	.89 [.82, .92]
Agreeableness	.86 [.84, .88]	.80 [.76, .83]	.59 [.45, .69]	.83 [.74, .87]
Neuroticism	.83 [.81, .85]	.80 [.76, .82]	.71 [.65, .76]	.82 [.72, .88]

95% confidence intervals are reported in brackets

#### Speeding in self-report

Participants who answer questions very quickly are unlikely to be giving sufficient effort or attention, hurting data quality. We defined speedy responders as those who answered questions on the BFI at an average rate of 1 s or less (Wood, Harms, Lowman, & DeSimone, 2017). Fourteen MTurk participants, 10 Prime Panels passed participants, and 24 Prime Panels failed participants met this criterion. After removing these speedy responders, we found that the MTurk workers (*M =* 3.46, *SD =* 3.50) responded faster, in average seconds per item, than either the Prime Panels passed (*M =* 4.71, *SD =* 4.42) or Prime Panels failed (*M =* 5.25, *SD =* 5.10) groups, *F*(2, 1177) = 16.24, *p <* .001. The two Prime Panels groups were not significantly different from each other (*p >* .14).

#### Missing data

Missing data were not part of our pre-registration, but missing data can be an indicator of data quality. Overall, the rate of missing data was low. Eighty percent of participants answered all 126 questions, and 97% of the participants had three of fewer missing responses. To see whether missing data varied by sample, we computed the percentage of missing responses for each participant. An ANOVA on the mean percentages of missing responses indicated a significant sample difference, *F*(2, 1225) = 22.97, *p <* .001. Participants who failed the screener on Prime Panels had more missing data (*M =* 0.19%, *SD =* 0.72) than either the MTurk (*M =* 0.001%, *SD =* 0.005) or the Prime Panels passed (*M =* 0.004%, *SD =* 0.18) groups, *p*s < .001. However, the MTurk and Prime Panels passed groups did not differ significantly, *p =* .55.

### Experimental studies as indices of data quality

For the three experiments reported next and the CRT, we did not anticipate between-group variation in effect size to be associated with participant demographics (e.g., group differences in age, political orientation, or religiosity), but instead treat effect size coefficients as indices of data quality. For the three tasks in which participants reported significant prior exposure—the trolley dilemma, the Asian disease task, and the Cognitive Reflection Task—we tested whether self-reported prior exposure lowered effect sizes. For all regressions in which the MTurk group is compared to another group, MTurk was entered as the reference group. For all regressions in which the Prime Panels passed group was compared to the Prime Panels failed group, Prime Panels passed was entered as the reference group.

#### Trolley dilemma

To analyze responses to the trolley dilemma, we collapsed “definitely yes” and “probably yes” responses into simply “yes,” and did the same for “definitely no” and “probably no” responses. Afterward, we observed that the experimental manipulation replicated across all samples (see Table 2).

**Table 2:** Response frequencies in the trolley dilemma study

Sample	Classic		Footbridge		
	No	Yes	No	Yes	*χ* ^2^
MTurk	21.8%	78.2%	69.5%	30.5%	(*N* = 446), 101.92^*^
Prime Panels passed	10.7%	89.3%	68.6%	31.4%	(*N* = 534), 189.77^*^
Prime Panels failed	20.2%	79.8%	62.2%	37.8%	(*N* = 248), 45.44^*^
MTurk naïve	14.5%	85.5%	65.6%	34.4%	(*N* = 173), 46.59^*^

^*^Statistically significant *χ*^2^ at the *p* < .001 level

Next, we compared the effects across conditions using logistic regression. Specifically, we regressed participant choice on experimental condition, whether participants came from the MTurk or Prime Panels samples (represented as dummy variables), and the interactions between condition and sample. Participants were more willing to sacrifice one person to save five others when doing so required turning the trolley than when pushing someone in front of it, *B* = 2.10, *p <* .001, 95% CI [2.53, 1.67]. This effect was qualified by a significant interaction between condition and sample, reflecting that participants who passed the screener on Prime Panels had bigger effect sizes than participants on MTurk, *B* = 0.80, *p =* .01, 95% CI [0.17, 1.43], and participants from Prime Panels who failed the screener, *B* = 1.03, *p <* .01, 95% CI [0.29, 1.76]. These interactions remained significant after several demographic covariates—age, education, marital status, and race—were added to the model (Prime Panels passed vs. MTurk, *B* = 0.95, *p* = .004, 95% CI [0.31, 1.59]; Prime Panels passed vs. Prime Panels failed, *B* = 1.14, *p* = .004, 95% CI [0.37, 1.92]). Finally, a separate regression analysis showed that prior exposure to the trolley dilemma significantly lowered the size of the effect, *B* = 2.30, *p* = .01, 95% CI [1.22, 4.34], and this was true even after controlling for the covariates mentioned above, *B* = 2.34, *p* = .01, 95% CI [1.22, 4.52].

Finally, as shown in the bottom row of Table 2, once non-naive MTurk participants were removed from the analyses, the differences between the MTurk and Prime Panels participants who passed the screener disappeared, *B* = – 0.48, *p >* .29, 95% CI [– 1.36, 0.40]. We omitted non-naive Prime Panels participants who failed the screener from this analysis and the analyses of non-naivete in all other experiments because previous exposure was assessed with a dichotomous yes-or-no question. Comparing the reported rates of non-naivete with those in Table 7 below shows that careless responding likely inflated non-naivete in the Prime Panels failed group, making it hard to draw meaningful conclusions about the effect of prior exposure within the sample.

#### Anchoring: Height of Mount Everest

**Fig. 1:**
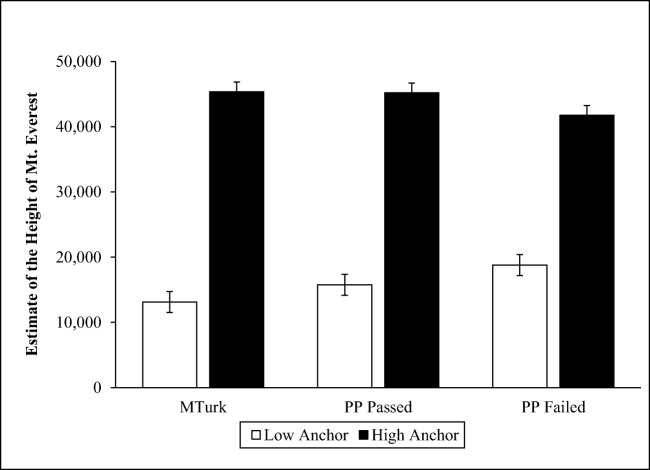
Estimates of the height of Mount Everest as a function of sample and anchor

We winsorized estimates greater than five standard deviations from the overall mean. The anchoring manipulation replicated across all samples (see Fig. 1).

We compared effect sizes across samples by regressing participants’ estimate of the height of Mount Everest on experimental condition, participant sample, and the interaction between condition and sample. We found a large effect of experimental condition, *B* = 32,236, *p* < .001, 95% CI [27,748, 36,724], and a significant interaction, indicating that the anchoring manipulation produced a larger effect in the MTurk sample than in the Prime Panels failed sample, *B* = 9,275, *p* = .02, 95% CI [16,830, 1,720]. Finally, a marginally significant interaction suggested that the anchoring manipulation also produced a larger effect in the Prime Panels passed sample than in the Prime Panels failed sample, *B* = 6,476, *p* = .08, 95% CI [13,812, -860]. After controlling for age, education, marital status, and race, these patterns remained the same (MTurk vs. Prime Panels failed: *B* = 8,385, *p* = .03, 95% CI [16,139, 632]) and marginally significant (Prime Panels passed vs. Prime Panels failed: *B* =6,386, *p* = .10, 95% CI [ 13,938, -1,164]).

Few participants reported prior exposure to the anchoring manipulation—less than 7% for both the MTurk and Prime Panels passed groups. Similar to the analyses that included non-naive participants, the anchoring manipulation yielded large effect sizes for both MTurk (*η*_p_^2^ = .20) and Prime Panels passed (*η*_p_^2^ = .22) participants who said they were naive to the task.

#### Asian disease

The framing effect in the Asian disease problem replicated in all samples (see Table 3). To compare the effects across samples, we again performed logistic regression using the same predictors used to analyze the trolley dilemma. We found a main effect of condition, *B* = 0.33, *p <* .001, 95% CI [0.24, 0.42], but none of the interaction terms were significant, indicating that the effects did not differ across samples. After controlling for age, education, marital status, and race, the main effects remained identical and the interactions remained not significant.

**Table 3:** Response frequencies in the Asian disease experiment

Sample	Positive frame		Negative frame	
	Response A	Response B	Response A	Response B
MTurk	73.7%^*^	26.3%	41.0%^*^	59.0%
Prime Panels passed	63.7%^*^	36.3%	37.1%^*^	62.9%
Prime Panels failed	65.1%	34.9%	42.6%	57.4%
MTurk naïve	73.5%^*^	26.5%	40.7%^*^	59.3%

^*^Statistically significant *χ*^2^ at the *p* < .05 level, relative to an H_o_ value of 50%

Although about 25% of MTurk workers reported prior exposure to the Asian disease problem, only 6.9% of the Prime Panels participants who passed the screener did the same. Effect sizes did not differ on the basis of prior exposure, *B* = 0.93, *p =* .82, 95% CI [0.49, 1.75].

#### Cognitive Reflection Test scores

There is a wide range of CRT scores in published research (Frederick, 2005; Toplak, West, & Stanovich, 2011), but findings show that students from top-ranked universities, such as MIT and Harvard, typically score toward the top of the distribution. MTurk workers far surpassed the typical range of scores on the CRT, performing similarly to students at top universities. A regression analysis indicated that sample explained about 28% of the variance in CRT scores, *F*(2, 1224) = 242.92, *p* < .001. MTurk workers answered more CRT questions correctly than either Prime Panels participants who passed the screener, *B* = 1.18, *p* < .001, 95% CI [1.30, 1.06], or Prime Panels participants who failed the screener, *B* = 1.42, *p* < .001, 95% CI [1.57, 1.27]. Prime Panels participants who passed the screener also scored higher than those who failed the screener, *B* = 0.24, *p* < .001, 95% CI [0.39, 0.09].

Additional analyses showed that self-reported prior exposure to the CRT significantly affected performance, *t*(979) = 12.35, *p <* .001, and this held true after controlling for covariates, *t*(979) = 12.39, *p <* .001.

### Sample representativeness

#### Demographics

The demographic characteristics of the MTurk, Prime Panels passed, Prime Panels failed, and ANES (a national probability sample) samples are presented in Tables, 4, 5, and 6. Table 4 presents basic demographics, including age, household income, marital status, children, race, ethnicity, and education level; Table 5 presents political orientation and political party affiliation; and Table 6 presents variables relating to religion.

**Table 4:** Basic demographics for the MTurk, Prime Panels, and ANES samples

	Sample			
	MTurk	Prime panels Passed	Prime panels Failed	ANES
Age				
18–29	31.2%	21.4%	22.6%	16.8%
30–39	38.5%	21.2%	31.5%	17.1%
40–49	16.1%	15.2%	15.2%	16.4%
50–59	10.0%	16.3%	13.5%	17.0%
60–69	3.8%	17.8%	12.2%	20.4%
70+	0.5%	8.1%	5.2%	12.2%
Annual household income				
<20k	11.9%	15.6%	24.2%	18.5%
20–39k	25.3%	26.1%	23.8%	22.1%
40–59k	20.9%	22.0%	13.9%	17.1%
60–79k	20.0%	14.3%	11.9%	11.7%
80–99k	10.5%	8.8%	11.9%	6.9%
100k+	11.4%	13.3%	14.3%	12.6%
Marital status				
Married	38.4%	49.0%	52.2%	47.3%
Never married	50.8%	34.0%	35.5%	34.6%
Previously married	10.8%	17.1%	12.2%	18.1%
Children				
Yes	40.0%	46.6%	50.6%	71.8%
Race				
White	75.3%	81.7%	70.6%	76.9%
Black	9.4%	7.3%	13.5%	12.7%
Asian	9.4%	4.1%	6.1%	5.7%
Other	5.8%	6.8%	9.8%	4.1%
Hispanic				
Yes	7.9%	7.1%	11.8%	17.8%
Highest degree				
No college degree	35.2%	48.3%	57.2%	73.0%
College degree	53.4%	39.1%	25.1%	16.8%
Postcollege degree	11.4%	12.6%	17.7%	10.2%

**Table 5:** Political views and party affiliation

	Sample			
	MTurk	Prime panels Passed	Prime panels Failed	ANES
Political party				
Republican	19.7%	29.3%	30.6%	29%
Democrat	44.2%	34.4%	37.1%	33%
Independent	30.9%	25.4%	17.1%	34%
Other	2.2%	1.9%	1.6%	4%
No preference	2.9%	9.0%	13.5%	–
Political views				
Extremely liberal	12.8%	8.3%	13.5%	14.8%
Liberal	27.4%	12.8%	14.7%	13.5%
Slightly liberal	16.0%	10.2%	9.0%	7.9%
Moderate	21.6%	38.5%	39.6%	28.1%
Slightly conservative	9.9%	11.1%	6.5%	10.4%
Conservative	8.1%	13.5%	12.7%	13.4%
Extremely conservative	4.3%	5.6%	4.1%	12.0%
Registered voter				
Yes	92.1%	86.2%	76.8%	87.2%

**Table 6:** Religiosity and religious practice for the MTurk, Prime panels, and ANES samples

	Sample			
	MTurk	Prime panels Passed	Prime panels Failed	ANES
Religion				
Buddhist	1.3%	1.5%	1.2%	0.8%
Christian	46.2%	59.4%	66.1%	58.9%
Muslim	0.2%	0.6%	1.6%	0.7%
Jewish	1.3%	2.6%	2.0%	2.5%
Hindu	0.7%	0.4%	2.4%	0.2%
Agnostic	18.8%	8.8%	2.9%	6.6%
Atheist	20.4%	7.9%	4.1%	6.8%
Other	7.0%	12.0%	11.8%	11.5%
Prefer not to say	4.0%	6.8%	7.8%	–
Religion importance				
Center of my entire life	7.8%	10.7%	13.5%	–
Very important	16.6%	31.7%	39.6%	–
Moderately important	17.0%	19.9%	20.8%	–
Not important at all, although I am religious	13.2%	11.3%	10.6%	–
I am not religious	45.3%	26.5%	15.5%	–
Born-Again Christian				
Yes	19.0%	28.9%	42.0%	29.4%
Belief in God				
Yes	58.5%	80.0%	88.3%	–
Frequency of prayer				
At least once a day	23.8%	41.9%	50.4%	–
Around once a week	13.0%	15.4%	19.8%	–
Around once a month	7.2%	8.5%	5.6%	–
A couple of times a year	8.5%	6.8%	7.7%	–
Less than once a year	4.7%	3.8%	4.4%	–
Never or not applicable	42.8%	23.7%	12.1%	–

Both groups of Prime Panels participants (*M*_pass_ = 45.58, *SD =* 16.56; *M*_fail_ = 41.87, *SD =* 15.26) were older than the MTurk participants (*M* = 36.49, *SD =* 11.08), but still younger than the ANES sample (*M* = 49.58, *SD =* 17.58). As expected, both groups of Prime Panels participants were less likely to have a college degree, less likely to be single, and more likely to have children, and also had a lower household income, than the MTurk participants. Participants from the ANES sample were more likely to have children and less likely to have a college degree than both the MTurk and Prime Panels participants, yet there were no differences in household income or marital status between the ANES and Prime Panels samples.

Compared to MTurk, both groups of Prime Panels participants were less liberal and less likely to identify as a Democrat, although they were still more liberal and more likely to identify as a Democrat than the ANES sample. Compared to MTurk, both the ANES and Prime Panels samples were more religious. Substantially larger proportions of both samples identified as a Christian, and specifically as a born-again Christian, and were less likely to identify as atheist or agnostic. The ANES and Prime Panels samples were similarly religious. Finally, Prime Panels participants also had substantially higher religiosity scores on self-reported religious beliefs (consulting God, belief in God, and importance of religion) than MTurk participants. These items were not included in the ANES study.

In sum, across a number of basic demographic variables, the Prime Panels sample reflected the nationally representative ANES sample more than the MTurk participants did.

#### Political attitudes

Participants answered several questions assessing their feelings toward a variety of minority groups and their opinions on a variety of political issues (see Tables S2–S11 in the supplemental materials). We examined these attitudes using two separate multivariate ANOVAs (MANOVAs) with pair-wise comparisons between samples. We omitted the Prime Panels failed group from these analyses because participant responses in this group were likely to be influenced by careless responding and represented a poor measure of underlying beliefs.

For the MANOVA assessing prejudice toward minority groups, we found significant sample differences, *F*_PillaisTrace_(24, 4286) = 8.23, *p* < .001. Specifically, across all questions, Prime Panels participants were more prejudiced toward minorities than MTurk participants, *F*(12, 954) = 2.10, *p* = .02. However, both the MTurk and Prime Panels samples were less prejudiced toward minorities than the ANES participants, *F*(12, 1616) = 7.61, *p* < .001, and *F*(12, 1703) = 12.28, *p <* .001, respectively.

As in the analysis of prejudice, there were overall differences between the samples for policy preferences, *F*_PillaisTrace_(16, 1924) = 5.47, *p* < .001. When comparing groups across all questions, Prime Panels participants were more conservative than MTurk participants, *F*(8, 789) = 8.03, *p* < .001. However, both the MTurk and Prime Panels samples were less conservative than the ANES participants [ANES vs. MTurk, *F*(8, 635) = 4.30, *p <* .001; ANES vs. Prime Panels, *F*(8, 491) = 3.96, *p* < .001].

### Experimental studies illustrating the importance of representativeness

For the two experiments reported next, we anticipated between-group variation in the effectiveness of the manipulation as a function of sample demographics (e.g., political orientation and religiosity).

#### With God on our side

**Fig. 2:**
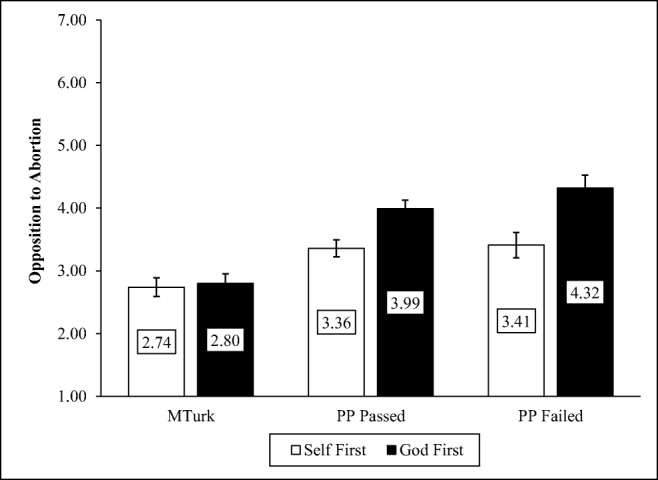
Opposition to abortion as a function of sample and condition

We analyzed responses to the “With God on our side” experiment using linear regression. Our analysis revealed a significant main effect of the experimental manipulation, indicating that when ignoring the sample, all participants were less opposed to abortion when thinking only about their own views than when first thinking about God’s views, *F*(1, 1224) = 14.52, *p* < .001. Next, there was also a significant main effect of sample, *F*(2, 1223) = 26.52, *p* < .001, with MTurk participants reporting less opposition to abortion than either of the Prime Panels groups, *t*s > 6.10, *p*s < .001. Most importantly for our purposes, there was a significant sample by manipulation interaction, *F*(5, 1220) = 14.96, *p* < .001 (see Fig. 2). Considering God’s opinion toward abortion before considering one’s own significantly increased opposition to abortion for both the Prime Panels participants who passed the screener, *B* = 0.57, *p* = .05, 95% CI [0.01, 1.14], and those who failed the screener, *B* = 0.85, *p* = .02, 95% CI [0.15, 1.55]. On MTurk, however, considering God's opinion about abortion prior to considering one's own did not significantly increase opposition to abortion, *B* = 0.06, *p* = .77, 95% CI [-0.34, 0.46]. There was no significant difference between the Prime Panels samples, *B* = 0.28, *p* = .42, 95% CI [– 0.40, 0.95].

#### Political equality experiment

**Fig. 3:**
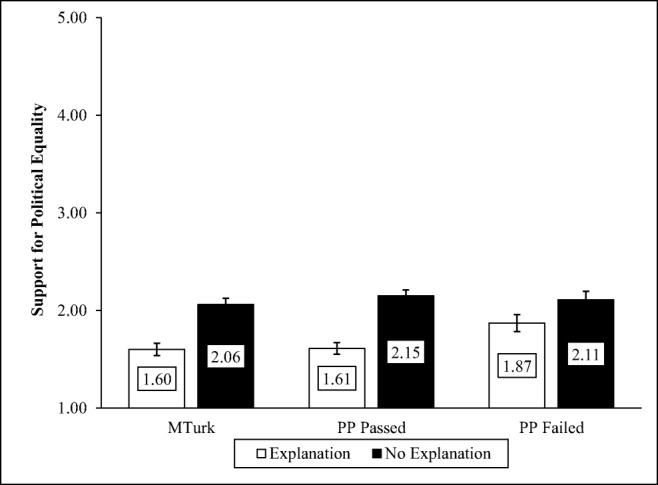
Support for political equality as a function of sample and whether or not equality was defined

A linear regression analysis showed a significant main effect of condition, *F*(1, 1225) = 69.23, *p* < .001, reflecting that regardless of sample, people were less likely to oppose political equality when an explanation was provided than when no explanation was provided, *B* = 0.46, *p* < .001, 95% CI [0.35, 0.56]. There was no main effect of sample, *F* = 2.34, *p* = .10, and only one significant interaction: The effect of the manipulation was larger for the Prime Panels passed than for the Prime Panels failed group, *B* = 0.30, *p* = .05, 95% CI [0.59, 0.01], *F*(5, 1221) = 15.61, *p* < .001. All other interactions were not significant, *t*s < 1.37, *p*s > .17 (see Fig. 3).

#### Non-naivete

MTurk workers reported more prior exposure to the CRT, trolley dilemma, and Asian disease tasks than participants who passed the screener on Prime Panels, *χ*^2^s(1, 980) > 63.52, *p <* .001 (see Table 7). MTurk workers also reported more prior exposure to the CRT and trolley dilemma than did Prime Panels participants who failed the screener, *χ*^2^(1, 694) > 42.92, *p <* .001. The samples did not differ in reported exposure to the Mount Everest anchoring task, with the exception of Prime Panels participants who failed the screener. We assume that the rate of reported exposure among Prime Panels participants who failed the screener is inflated by noise and careless responding.

**Table 7:** Reported rates of non-naivete

Sample	Task			
	Cognitive reflection task	Trolley dilemma	Asian disease	Mt. Everest
MTurk	78.7%	61.2%	25.3%	5.6%
Prime Panels passed	19.1%	11.0%	6.9%	6.6%
Prime Panels failed	29.8%	23.8%	21.0%	22.2%

## Discussion

The goal of this study was to explore whether online panels can serve as a plausible alternative to MTurk for participant recruitment in the social and behavioral sciences. Unlike previous comparisons between MTurk and online panels (Heen, Lieberman, & Miethe, 2014; Kees et al., 2017), we employed and tested a prescreening measure that is standard on Prime Panels and serves a similar purpose on Prime Panels as MTurk’s reputation mechanism. We found that a substantial portion of respondents from Prime Panels failed the screener. However, responses from the Prime Panels participants who passed the screener compared favorably to a sample recruited from MTurk in terms of data quality, demographic composition, and participant naivete.

### Data quality and non-naivete

Once participants from Prime Panels passed an initial screener, the quality of their data was similar to that observed on MTurk. Prime Panels participants passed attention checks at a high rate, though not quite as high as MTurk workers who are unusually good at passing data quality checks (see Hauser & Schwarz, 2016; Thomas & Clifford, 2017). Scale reliabilities were equivalently high, and, in four experiments predicted to be insensitive to demographic differences, we found effect sizes identical to those observed on MTurk. In several experiments, the effect sizes were higher on Prime Panels than on MTurk. Taken together, these results show that participants recruited through Prime Panels were about as attentive to the stimulus materials as MTurk workers.

We observed that familiarity with the stimuli from this study was significantly higher on MTurk than Prime Panels, a difference that might be expected given the number of behavioral scientists who use MTurk. Indeed, in three out of the four measures of non-naivete, prior exposure was higher on MTurk than Prime Panels. For the CRT specifically, 78% of MTurk participants reported prior exposure, over 50% higher than Prime Panels. Self-reported prior exposure was associated with effect sizes of experimental manipulations and measures. The effect sizes of the trolley dilemma were lower for participants who reported prior exposure, and CRT scores were significantly higher for those who were not naïve to the task. No differences for the Asian disease experiment were observed on the basis of self-reported prior exposure. It is likely that some dependent measures are more influenced by prior exposure than others, and that self-reported dichotomous measures of exposure are imperfect measures of actual exposure (Chandler et al., 2015). To the extent that self-reported exposure provides information about population level familiarity with specific measures, our data clearly show that Prime Panels participants were less familiar with common experimental manipulations than MTurk samples and that familiarity was associated with effect sizes of experimental manipulations and performance measures (see Chandler et al., 2015).

At the same time, the degree of naivete of MTurk samples depends heavily on the selection criteria used in a study: researchers can take steps to limit the experience of MTurk workers recruited for their studies (for a discussion see Robinson et al., 2019). It is possible that excluding more active MTurk workers may lead effect sizes observed on MTurk to more closely resemble those observed on Prime Panels.

### Sample composition

Prime Panels participants were more diverse than MTurk participants and possessed demographic characteristics that more closely resembled the US population as a whole. In particular, the age of Prime Panels participants was much closer to the average for the US population than that of MTurk workers. As an older sample, it is not surprising that Prime Panels participants were also more religious, more likely to be married and to have children, less likely to have a college degree, and more politically conservative than MTurk participants.

The demographic characteristics of Prime Panels allow researchers to recruit samples that would be difficult or impossible to recruit using MTurk alone. If, for example, researchers wanted to obtain a very large sample (i.e., thousands of participants) that closely matched the demographics of the US, or a large sample matched to the US population on a demographic characteristic like wealth (see Davidai, 2018) or religion, it’s unlikely the sample could be obtained on MTurk. Further, and of particular relevance to social scientists, Prime Panels could be especially useful for recruiting groups that are hard to sample on MTurk or from college campuses, such as Republicans or senior citizens. Because only 3.3% of MTurk workers are above age 60, there are simply not enough workers to sample from in the MTurk pool. In addition, the scarcity of older workers on MTurk raises important questions about whether the older adults working on MTurk differ in important ways from older adults not working on MTurk—a concern that is less relevant for younger workers (Huff & Tingley, 2015). On Prime Panels, over 23% of the participants were above age 60. This suggests that there could be millions of older participants in online research panels, making these panels an ideal supplement to MTurk for online research with the elderly or across the lifespan.

As our study illustrated, although demographic representativeness is not important for many studies (Coppock, Leeper, & Mullinix, 2018), it matters when samples differ in the characteristics necessary to produce a specific phenomenon. In the with God on Our Side experiment, we observed that thinking about God’s attitudes toward abortion caused Prime Panels participants to become more opposed to abortion, a finding reliably observed in nationally representative samples. The same manipulation, however, did not influence MTurk workers who are largely not religious. This finding is consistent with other work showing that the with God on Our Side experiment replicates in nationally representative samples but not on MTurk (Mullinix et al., 2015). Furthermore, this finding points to the importance of considering whether the demographics of one’s intended sample might interact with the phenomena under investigation (see Sears, 1986).

### Prescreening to improve data quality in online panels

Our data provide strong evidence that the inclusion of a prescreen in online research panels is both necessary and sufficient to increase data quality to acceptable levels. Participants who failed the screener had unacceptably low internal reliability scores on survey questions, low pass rates on attention checks, and lower effect sizes on experimental manipulations. On three of the five personality measures, for example, the internal reliability for Prime Panels participants who failed the screener were below .6. For Prime Panels participants who passed the screener, internal reliabilities were comparable to published norms. Our data are consistent with previous studies on other online panel platforms in showing that over 30% of respondents are inattentive and not suitable for the purposes of most academic research (Thomas & Clifford, 2017). However, here we showed that including a basic screener can substitute for the reputation mechanism that operates on MTurk. On Prime Panels, all participants must complete screeners like these prior to attempting a study so that the initial dataset is not contaminated with low-quality responses. In principle, similar validated screeners can be added by researchers recruting participants in other research contexts, including non-web surveys.

### The structure of Prime Panels

While MTurk is a crowdsourced platform, Prime Panels works like an exchange. Studies launched by researchers are bid on by suppliers, who compete to provide the sample. Because most academic studies sample a few hundred participants from the general population, studies on Prime Panels are often provided by a single supplier. However, if the supplier that wins a study bid is not able to send a sufficient number of participants to meet a study’s desired sample, other suppliers can make additional bids to help supplement the sample. The purpose of having multiple suppliers and multiple bids is to extend the size of the participant pool, to make finding hard-to-reach groups feasible, and in many cases, to reduce price. When a study targets a specific, hard-to-reach group (e.g., African Americans below age 25) and the sample is large (e.g., 5,000), it is unlikely that any single provider, including MTurk, would be able to recruit sufficient numbers of participants to fill the quota. In such cases, multiple providers are typically needed in order to recruit enough participants.

Sampling from multiple suppliers does not limit the generalizability of results, for three reasons. First, whereas some demographic differences may exist across different sample providers, all suppliers are more closely matched to the general US population than MTurk in terms of key demographic variables such as age, education, and political affiliation. Previous studies on Prime Panels showed demographic representativeness similar to what we have (e.g. Ballew et al, 2019; Job et al., 2018; Davidai, 2018; Deri, Davidai, & Gilovich, 2017; Waggoner, 2018). Second, the screener used in this study, and that is similar to standard screening methodology used by Prime Panels, ensures that differences in data quality across suppliers will manifest as different pre-study exclusion rates rather than differences in study data quality. Finally, researchers can apply sampling quotas or use a census-matched template, both of which ensure that the demographics of any study closely match that of the population.

### Other considerations when deciding between MTurk and online research panels

In this study, we showed that online research panels can serve as an alternative to MTurk and can offer some distinct advantages over the MTurk platform. In addition to sample diversity, online research panels offer significant operational advantages over MTurk when recruiting rare groups. Whereas MTurk allows for targeted recruitment of specific population segments, online research panels allow for more complex targeting. For example, Prime Panels allows researchers to target participants in specific cities and zip codes, to match the sample to the census on multiple demographics, and to use prescreens that can cull participants on the basis of demographics and behavior that have not been previously profiled (e.g., Davidai, 2018). Online research panels also provide access to populations in dozens of countries around the world, whereas MTurk primarily provides access to samples in the United States, Canada, and India.

Both MTurk and online research panels make it possible to collect hundreds or even thousands of responses within hours or just a few days. Despite this similarity, MTurk and online panels have very different ecosystems and participant cultures. Specifically, MTurk gives researchers more control over various study settings and allows researchers to communicate directly with participants. Perhaps the biggest difference between the platforms is in how participants are compensated. On MTurk, researchers have complete control over wages, including the ability to pay bonuses at their discretion and fees paid to Amazon are a percentage of the payment amount. In online research panels, however, researchers pay a vendor-specified cost per complete that includes fees and the compensation that participants ultimately receive. The compensation offered by online research panels varies considerably across sample providers, in terms of both type and amount. Compensation may typically include cash, gift cards, reward points, or a donation to a charity chosen by the participant.

For simple studies, the costs of running a study on MTurk and Prime Panels are often comparable. For example, a 15-min study on MTurk might cost $1.80 per participant (including MTurk’s fee of 20%), assuming that participants are paid $6 per hour. On Prime Panels a similar study would cost $1.50 per participant (including all fees). Because compensation on MTurk is left solely to the researcher, these costs can remain static regardless of the complexity of the design. In online research panels, however, cost depends on the difficulty of obtaining the desired sample, and the target population incidence rate, speed of data collection, and sample size can all increase costs.

### Future research

MTurk workers are well understood in terms of their demographics, attitudes, and behavior, such as participation rates in typical studies, sample composition across times of day, and follow-up rates in longitudinal studies. Much less is known about online research panels in terms of both their demographics and behavior. Whether longitudinal research can be carried out successfully, what the turnover rate of participants in the subject pool is, and how sample composition varies across times of day and days of the week are questions that have not been fully investigated. Significant research will be required in order to fully understand the value of online research panels for research in the social and behavioral sciences, as well as their potential to supplement or replace MTurk as a source of participant recruitment.

From what is already known about online research panels, data quality is the greatest concern. Our study shows that prescreening methods that are standard on Prime Panels can improve data quality. Panel providers differ in their use of prescreens, and significantly more research will be required in order to develop better screening methods and to understand how screening impacts studies of various complexity, such as open-ended research and research that requires extensive engagement. At the same time, the research conducted in this study suggests that online panels may provide a viable alternative for many types of research studies, provided that sufficient attention is allocated to using effective screening methods. Future studies should explore in more depth the specific ways in which online research panels and MTurk can be used to enhance online recruitment of participants for research in the social and behavioral sciences.

### Conclusion

Online participant recruitment has become an important part of the social and behavioral sciences. The most commonly used platform today, Amazon Mechanical Turk, generally produces high-quality data but has limitations in terms of the size and diversity of its sample that necessitate looking for supplemental sources of participants. Online research panels, such as those accessed through Prime Panels, offer a more diverse and nationally representative sample than MTurk. Furthermore, many of the data quality issues associated with online research panels can be addressed with the use of a prescreening methodology for respondent attentiveness. The potential advantages of online research panels, combined with the increasing ease of using them, suggest that they are worth considering as a tool to recruit large, diverse, or hard-to-reach samples.

## Electronic supplementary material

ESM 1 (DOCX 30 kb)
