# Supplementary material for: Online panels in social science research: Expanding sampling methods beyond Mechanical Turk
Source: Behav Res Methods. 2019 Sep 11;51(5):2022–38. doi: 10.3758/s13428-019-01273-7 (PMC6797699; doi:10.3758/s13428-019-01273-7)
Supplement: Supplementary file 1 — (DOCX 30 kb) [file 13428_2019_1273_MOESM1_ESM.docx]

Section I. Tables

Table S1

*Alpha coefficients for the dimensions of the BFI.*

|  | Sample | | | | |
| --- | --- | --- | --- | --- | --- |
| Dimension of BFI | MTurk | Prime Panels  passed | Prime Panels  failed | Buhrmester et al., (2011) | Litman et al., (2015) |
| Openness | .87 | .78 | .71 | .79 | .83 |
| Conscientiousness | .90 | .83 | .72 | .77 | .85 |
| Extraversion | .90 | .83 | .59 | .86 | .89 |
| Agreeableness | .86 | .87 | .72 | .77 | .83 |
| Neuroticism | .88 | .85 | .70 | .85 | .82 |

*Note:* Buhrmester et al., (2011) and Litman et al., (2015) were both studies conducted with Mturk workers.

Table S2

*Prejudice toward minority groups*

How well does the word “XXX” describe most members of each group?

|  | Sample | | |  |  |  |
| --- | --- | --- | --- | --- | --- | --- |
| Lazy | MTurk | Prime Panels | ANES | MTurk vs. Prime Panels | MTurk vs. ANES | Prime Panels vs. ANES |
| Blacks | 1.79 (1.17) | 1.76 (1.16) | 2.35 (1.32) | .72 | <.001 | <.001 |
| Whites | 1.64 (0.96) | 1.70 (1.08) | 2.03 (1.08) | .39 | <.001 | <.001 |
| Hispanics | 1.50 (0.93) | 1.54 (0.96) | 1.83 (1.12) | .57 | <.001 | <.001 |
| Muslims | 1.56 (1.00) | 1.58 (1.01) | 1.93 (1.18) | .86 | <.001 | <.001 |
| Violent |  |  |  |  |  |  |
| Blacks | 2.13 (1.33) | 2.06 (1.31) | 2.60 (1.30) | .40 | <.001 | <.001 |
| Whites | 1.87 (1.08) | 1.88 (1.14) | 2.25 (1.14) | .89 | <.001 | <.001 |
| Hispanics | 1.88 (1.11) | 1.89 (1.20) | 2.16 (1.12) | .90 | <.001 | <.001 |
| Muslims | 2.22 (1.42) | 2.10 (1.35) | 2.79 (1.45) | .17 | <.001 | <.001 |

*Note:* Whites were not included in the MANOVA reported in the main text, but we include them here as a point of comparison for the minority groups.

Table S3.

*How much discrimination is there in the United States today against each of the following groups?*

|  | Sample | | |  |  |  |
| --- | --- | --- | --- | --- | --- | --- |
| Group | MTurk | Prime Panels | ANES | MTurk vs. PP | MTurk vs. ANES | PP vs. ANES |
| Blacks | 3.57 (1.09) | 3.57 (1.18) | 3.29 (1.16) | .99 | <.001 | <.001 |
| Whites | 1.92 (0.94) | 2.33 (1.23) | 2.26 (1.18) | <.001 | <.001 | .26 |
| Hispanics | 3.21 (1.05) | 3.22 (1.16) | 2.99 (1.05) | .87 | <.001 | <.001 |
| Gays and Lesbians | 3.45 (1.06) | 3.54 (1.18) | 3.18 (1.16) | .21 | <.001 | <.001 |
| Women | 2.91 (1.05) | 2.88 (1.10) | 2.74 (1.02) | .64 | <.01 | .01 |
| Men | 1.83 (0.94) | 2.05 (1.12) | 2.03 (1.13) | <.01 | <.01 | .75 |
| Transgender People | 3.80 (1.09) | 3.72 (1.20) | 3.45 (1.22) | .31 | <.001 | <.001 |
| Muslims | 3.96 (1.07) | 3.89 (1.17) | 3.64 (1.24) | .33 | <.001 | <.001 |
| Christians | 2.03 (1.08) | 2.53 (1.27) | 2.57 (1.33) | <.001 | <.001 | .60 |

Table S4

*In general, do the police treat whites better than blacks, treat blacks better than whites, or treat them both the same?*

| Sample | | |  |  |  |
| --- | --- | --- | --- | --- | --- |
| MTurk | Prime Panels | ANES | MTurk vs. PP | MTurk vs. ANES | PP vs. ANES |
| 3.73 (1.17) | 3.32 (1.25) | 3.35 (1.26) | <.001 | <.001 | .66 |

Table S5

*Do you favor, oppose, or neither favor nor oppose allowing universities to increase the number of black students studying at their schools by considering race along with other factors when choosing students?*

| Sample | | |  |  |  |
| --- | --- | --- | --- | --- | --- |
| MTurk | Prime Panels | ANES | MTurk vs. PP | MTurk vs. ANES | PP vs. ANES |
| 3.78 (1.95) | 3.79 (2.00) | 3.56 (2.00) | .92 | .08 | .05 |

Table S6

*Do you favor, oppose, or neither favor nor oppose requiring employers to pay women and men the same amount for the same work?*

| Sample | | |  |  |  |
| --- | --- | --- | --- | --- | --- |
| MTurk | Prime Panels | ANES | MTurk vs. PP | MTurk vs. ANES | PP vs. ANES |
| 6.14 (1.52) | 6.27 (1.30) | 5.94 (1.57) | .21 | .01 | <.001 |

Table S7

*Do you think business owners who provide wedding-related services should be allowed to refuse services to same-sex couples if same-sex marriage violates their religious beliefs, or do you think business owners should be required to provide services regardless of a couple's sexual orientation?*

| Sample | | |  |  |  |
| --- | --- | --- | --- | --- | --- |
| MTurk | Prime Panels | ANES | MTurk vs. PP | MTurk vs. ANES | PP vs. ANES |
| 1.45 (0.50) | 1.50 (0.50) | 1.55 (0.50) | .09 | <.001 | .11 |

Table S8

*Do you favor, oppose, or neither favor nor oppose the death penalty for persons convicted of murder?*

| Sample | | |  |  |  |
| --- | --- | --- | --- | --- | --- |
| MTurk | Prime Panels | ANES | MTurk vs. PP | MTurk vs. ANES | PP vs. ANES |
| 3.39 (1.37) | 3.65 (1.28) | 3.60 (1.33) | <.01 | .01 | .51 |

Table S9

*When people from other countries legally move to the United States to live and work, is this generally good for the U.S., generally bad for the U.S., or neither good nor bad?*

| Sample | | |  |  |  |
| --- | --- | --- | --- | --- | --- |
| MTurk | Prime Panels | ANES | MTurk vs. PP | MTurk vs. ANES | PP vs. ANES |
| 5.34 (1.51) | 5.22 (1.60) | 5.04 (1.71) | .25 | .04 | <.01 |

Table S10

*How worried are you that the United States will experience a terrorist attack in the near future?*

| Sample | | |  |  |  |
| --- | --- | --- | --- | --- | --- |
| MTurk | Prime Panels | ANES | MTurk vs. PP | MTurk vs. ANES | PP vs. ANES |
| 2.80 (1.20) | 3.33 (1.18) | 3.23 (1.33) | <.001 | <.001 | .17 |

Table S11

*Some people think that the way people talk needs to change with the times to be more sensitive to people from different backgrounds. Others think that this has already gone too far and many people are just too easily offended. Which is closer to your opinion?*

| Sample | | |  |  |  |
| --- | --- | --- | --- | --- | --- |
| MTurk | Prime Panels | ANES | MTurk vs. PP | MTurk vs. ANES | PP vs. ANES |
| 2.55 (1.09) | 2.52 (1.12) | 2.85 (1.10) | .75 | <.001 | <.001 |

Section II. Supplementary Results

**Data Quality Measures**

As measures of data quality, we conducted exploratory analyses on variables that measured the device participants used to complete the study, the browser they used to complete the study, and the number of times they tried to reattempt the survey. Survey reattempts are a common practice whereby participants can try to get around attention manipulation checks. For example, using Incognito mode on Chrome allows participants to click on a survey link multiple times without being blocked even if Qualtrics’s “prevent ballot box stuffing” feature is turned on. To catch reattempts, we automatically captured each participant’s unique platform ID each time they entered the survey.

Participants on MTurk used a desktop computer much more often (95%) than participants on Prime Panels (69%), χ^2^ (1, 1227) = 109.80, *p* < .001. Prime Panels participants who passed the screener were only slightly more likely (70.6%) to use a desktop computer than Prime Panels participants who failed the screener (64.4%), χ^2^ (1, 781) = 3.04, *p* = .08.

For all three samples, Google Chrome was the most common internet browser (MTurk = 76%, Prime Panels passed = 57%, and Prime Panels failed = 63%). Noticeably more Prime Panels participants used Internet Explorer (Prime Panels passed = 16%, and Prime Panels failed = 14%) than MTurk participants (5%) and the same thing was true for Safari (MTurk = 4%, Prime Panels passed = 17%, and Prime Panels failed = 14%). Approximately 10% to 15% of each sample used Firefox (MTurk = 15%, Prime Panels passed = 10%, and Prime Panels failed = 9%).

In this study, we did not find any evidence of survey reattempts by participants on MTurk or Prime Panels.

**Squared Discrepancy Procedure**

We examined the consistency of participants’ responses across the ten pairs of antonymous traits by calculating a Squared Discrepancy Score (SDS). SDS is a measure of participant-level response-consistency to reversed Likert-type items (see Litman, Robinson, & Rosenzweig, 2015, for a detailed description). This measure assumes that people should provide logically consistent responses to antonymous traits, endorsing one, but not the other. With five response options (1 - *strongly agree* to 5 - *strongly disagree*), the discrepancy between each item and its reversed form could range from 0 and 4. For example, if a respondent strongly agreed to both being organized and being disorganized, the discrepancy score would be 4, the maximum. Discrepancies for each pair are squared to place greater emphasis on highly inconsistent responses. This means a respondent who strongly agreed to both being talkative and not being talkative would have a squared discrepancy score of 16, while a participant who strongly agreed to being talkative but merely disagreed (instead of strongly disagreed) to not being talkative would have a squared discrepancy score of 1.

The SDS is the sum of squared discrepancy scores from all ten pairs of questions, converted to a percentage that ranges from 0% to 100% and then reversed so that a score of 0% indicates *maximally inconsistent performance* and a score of 100% indicates *maximally consistent performance*. A Monte Carlo simulation indicated that truly random responses produce an SDS of 70 (*SD* = 7; Litman et al., 2015). We define consistent responders as those who demonstrate consistency scores > 2 standard deviations above what would be expected by a random responder.

Squared Discrepancy Scores were largely consistent with the reliability coefficients in showing that: 1) MTurk workers performed somewhat better than Prime Panels participants who passed the screener—although MTurk workers in this study provided very consistent scores that may be unusually high when compared to non-MTurk data and MTurk data from previous years, 2) Prime Panels participants who failed the initial screener tended to provide inconsistent responses, and 3) Prime Panels participants who passed the screener performed at a high level, comparable to other MTurk studies. Specifically, one-sample t-tests compared the squared discrepancy scores to a test value of 84. This test value corresponds to 2 SDs above chance performance as modeled by Monte Carlo simulations (see Litman et al., 2015). Both MTurk participants and Prime Panels participants who passed the initial screener had Squared Discrepancy Scores that were significantly higher than 84: MTurk, *t*(473) = 29.5, *p* < .001; Prime Panels, *t*(531) = 15, *p* < .001. Meanwhile, Prime Panels participants who failed the screener had Squared Discrepancy Scores that were significantly below the test value of 84 *t*(241) = -2, *p* < .05.
